# Factors Influencing Menarcheal Age: Results From the Cohort of Tehran Lipid and Glucose Study

**DOI:** 10.5812/ijem.16130

**Published:** 2014-06-10

**Authors:** Fahimeh Ramezani Tehrani, Parvin Mirmiran, Roya Gholami, Nazanin Moslehi, Feriedon Azizi

**Affiliations:** 1 Reproductive Endocrinology Research Center, Research Institute for Endocrine Sciences, Shahid Beheshti University of Medical Sciences, Tehran, IR Iran; 2 Obesity Research Center, Research Institute for Endocrine Sciences, Shahid Beheshti University of Medical Sciences, Tehran, IR Iran; 3 Endocrine Research Center, Research Institute for Endocrine Sciences, Shahid Beheshti University of Medical Sciences, Tehran, IR Iran

**Keywords:** Body Mass Index, Longitudinal Studies, Menarche, Maternal Educational Status.

## Abstract

**Background:**

Menarche is considered as a milestone in the women’s reproductive life. Most existing studies on factors influencing menarcheal age had cross-sectional designs and their finding were controversial.

**Objectives:**

We aimed to determine some factors affecting the age at menarche in a cohort study with an average of ten-year follow-up; the study was conducted within the framework of Tehran Lipid and Glucose Study (TLGS).

**Materials and Methods:**

For the purpose of the present study, we recruited all the females aged 12 to 18 years participated in TLGS whose menarche had not begun at the initiation of the study, but occurred during their follow-up. The effect of premenarcheal status of various factors including socioeconomic and anthropometric parameters, physical activity, energy expenditure, and exposure to tobacco smoke on menarcheal age was explored.

**Results:**

The mean of age at menarche was 13.06 ± 1.24 years. There were significant statistical associations between age of the participants’ mothers at menarche (r = 0.66, P < 0.001), maternal education (r = -0.04, P = 0.002), and body mass index (BMI) before menarcheal (r = 0.25, P = 0.027) with age at menarche. There was no significant correlation between age at menarche, with either of maternal employment, premenarcheal physical activity, energy expenditure, and passive smoking.

**Conclusions:**

Among various factor influencing menarcheal age, premenarcheal BMI is modifiable, and considering its significance, could prevent early or late menarches.

## 1. Background

The first menstrual period is called menarche (1), which is a vital event in the development of female adolescents’ puberty. Despite other pubertal changes that are gradual and continuous, menarche is a distinct event with a sudden onset. The timing of menarche is an important determinant of population size, reproductive performance, and other chronic outcomes such as cancers of the reproductive organs (2); moreover, it is an important factor in health planning (3). Menarche is affected by genetic factors, race, environmental conditions, nutrition, physical activity, geographic location, urban or rural residence, health status, psychological factors, blindness, body mass index (BMI), family size, socioeconomic status, parental educational level, occupation of parents, loss of parents, child sexual abuse, physical stress, tea consumption, and passive smoking (1, 4-12). Various studies indicate that the average age of menarche has decreased significantly in the last 100 years, which there has been a secular (time-related) trend towards an earlier onset of menarche in most developed countries, with a decline of two to three months per decade in Europe and the United States (2, 3). In recent years, such a decline has also been observed in developing countries (3).

Several regional and national studies on the timing of menarche have been conducted in Iran (1, 13-16). The mean age at menarche in Iranian girls born between 1985 and 1989 was 12.5 years (1). The age at menarche was assessed in two national Iranian surveys conducted in 1990 and 2008 among girls across the country with sample sizes of 88220 and 10228 participants, respectively. According to their results, the national average age at menarche was 13.86 ± 1.51 and 13.26 ± 1.61 years in 1990 and 2008, respectively.

Based on these findings, there has been a secular trend towards an earlier onset of menarche in Iranian females with a decline of one month per four years (11). A study of Tehranian female born during third to ninth decades of previous century indicated a decrease in the mean age at menarche from 13.88 years to 12.98 years (17). Another study of mothers and daughters with at least 25 years difference in birth date showed that the mean age of menarche in daughters and mothers was 13.2 ± 1.4 and 13.6 ± 1.5 years, respectively, indicating a decrease in age of menarche from one generation to the next (18). As shown by the 2006 national survey, girls aged 10-19 years constitute a large percentage of the total Iranian female population (19). Unfortunately, the factors influencing age at menarche have not yet been studied at a national level and the findings of regional studies documented are inconsistent (20). In addition, given the importance of the onset of menstruation in public health, there is limited data available on the effects of various factors on the menarcheal age.

## 2. Objectives

We aimed to determine the factors influencing menarcheal age.

## 3. Materials and Methods

This longitudinal study was conducted within the framework of Tehran Lipid and Glucose Study (TLGS), initiated in 1998 with the aim of determining the prevalence of non-communicable diseases risk factors. A total of 15005 females aged ≥ 3 years selected from a geographically defined population using multistage cluster sampling, were recruited after obtaining written consent from them or their guardians. Demographic and lifestyle variables as well as data on family size, parents occupation as well as educational level, menarcheal age of mother, and the medical and reproductive histories were collected through face-to-face interviews by trained interviewers at baseline and once in every three years. The follow-up included a general physical examination as well as height and weight measurements (21).

Among the 1462 girls (12, 13, 17-21) who participated in TLGS, 402 met our eligibility criteria. Those with missing data and those who experienced menarche before initiation of the study were excluded. The recruited girls were evaluated once every 3 years at follow ups until the onset of menstruation; if menarche occurred during the follow-up, the exact menarcheal age was documented and BMI, basal metabolic rate (BMR), physical activity, and exposure to tobacco smoke before the menarche were calculated. Height was measured by a tape measure in a standing position without shoes while the shoulders were in a normal position. Weight was measured with the women minimally clothed, without shoes, using a digital scale and the value was rounded to the nearest 100 g. BMI (kg/m^2^) was calculated as weight (kg) divided by the height square (m^2^). According to BMI and by employing the standard quantities recommended by Kelishadi, participants were categorized as follows: Underweight, BMI < 15th percentile; normal weight, BMI between 15th to 85th percentile; overweight, BMI between 85th 95th percentile; and obese, BMI > 95th percentile. These percentiles were obtained according to anthropometric parameters of all 12-19 year old girl participants in the TLGS. BMR was calculated using the Harris-Benedict formula:

BMR = 665 + [9.6 × weight (kg)] + [1.8 × height (cm)] – [4.7 × age (year)].

Physical activity was calculated according to those activities performed more than ten times per year during the leisure hours; these activities included running, fighting sports or martial arts, gymnastic, endurance sports, football, handball, basketball, volleyball, cycling, tennis, bodybuilding/weight lifting, swimming/diving, wrestling, skiing, skating, climbing, walking, and dancing/limbering with music. Activities during school hours were not included. We weighted each activities based on its intensity and the final physical activity score was calculated based on MET unit (1 MET is equal to 3.5 mL of consumed oxygen per minute for 1 kg of body weight).

Exposure to tobacco smoke was calculated using the following formula: 

Number of smokers at home × hours of daily exposure to tobacco smoke × days of weekly exposure to tobacco smoke.

### 3.1. Statistical Analysis

Continuous variables were tested for normality using the one-sample Kolmogorov-Smirnoff test; Pearson correlation analysis was used to determine the association between the menarcheal age with BMI and menarcheal age of their mothers. Multiple linear regression analysis was utilized to evaluate the predictors of menarcheal age as a dependent variable, using maternal menarcheal age, BMI, calorie intake, exposure to tobacco smoke, and physical activity as predictive variables. Analysis of variance (ANOVA) and independent samples t test were used to compare the age at menarche in different BMI groups. Data analysis was performed by SPSS 15.0 PC package (SPSS Inc., Chicago, IL, US). Statistical significance was determined at P < 0.05.

## 4. Results

There was no significant difference between demographic indicators and mean menarcheal age of girls in our study and those excluded from the study because of missing data (data not shown). Overall, mean menarcheal age was 13.06 ± 1.24 years and, based on our findings, the age of menarche in 78.4% of cases ranged from 12 to 14 years. Mean BMI was 20.46 ± 3.85 kg/m^2^ and mean maternal menarcheal age was 13.15 ± 1.23 years (Table 1). Among the participants, 77.7% had normal BMI; however, 4.3%, 10.4%, and 7.7% were underweight, overweight, and obese, respectively. There was only a statistically significant difference between menarcheal age of underweight group (14.4 ± 1.1) in comparison to all three other groups, namely, the normal, overweight, and obese (13.0 ± 1.2, 12.8 ± 1.3, and 12.8 ± 1.2, respectively). There was a significant negative correlation between menarche and BMI (r = -0.13, P = 0.012). The mean of menarcheal age of the girls whose mothers had primary education levels was significantly lower than those whose mothers’ had higher levels of education (12.9 vs. 13.5 years; p = 0.01); however, there was no significant association between menarcheal age of girl and the mother’s occupation, exposure to tobacco smoke, physical activity, or calorie intake (Table 2).

There was a significant statistical association between the menarcheal age of girls with maternal menarcheal age and the family size (r = -0.676, P < 0.001; and r = -0.15, P = 0.002, respectively), indicating that with increased maternal menarcheal age and family size, the menarcheal age of daughters would be increased. Table 3 shows results of the multiple linear regression analysis using daughter’s menarcheal age as the dependent variable and predictive variables including educational as well as employment status of mothers, family size, maternal menarcheal age, BMI, calorie intake, and exposure to tobacco smoke are shown in Table 3. Variables that had the ability to predict the age at menarche and remained in the final model were BMI, maternal educational level, and maternal menarcheal age (Table 3).

**Table 1.** Characteristics of the Study Participants ^a^

Characteristics	Mean ± SD	No (%)
**Menarcheal Age of Daughter, y**	13.06 ± 1.24	-
**Menarcheal Age of Mother, y**	13.15 ± 1.23	-
**Maternal Educational Level, %**	-	-
Primary Education	-	281 (69.9)
Secondary Education Or Higher Degree	-	91 (22.6)
**Number Of Family Members**	-	5.9 (1.6)
**Premenarcheal Characteristics**		
**BMI, Kg/m^2^**	20.46 ± 3.85	-
**Physical Activity^a^, min/wk**	199 ± 765	-
**Calorie Intake^b^, kcal**	1350 ± 113	-
Exposure to Tobacco Smoke, person-hour/wk	-	0

^a^ Abbreviations: SD, standard deviation; BMI, body mass index.

^b^ There were no data concerning girls who had their menarche between phases I and II; therefore, these girls were omitted.

**Table 2.** A Comparison of Menarcheal Age Based on Different Variables

Variables	Mean of Menarcheal, age
**Body Mass Index, kg/m2 (P = 0.01)^a^**	
Underweight	14.38 ± 1.08
Normal	12.99 ± 1.18
Overweight	12.79 ± 1.30
Obese	12.83 ± 1.20
**Maternal Educational Level (P = 0.03)**	
Primary Education	12.94 ± 1.24
Secondary Education Or Higher Degree	13.47 ± 1.19
**Physical Activity** ^**b**^	
Active	13.08 ± 1.29
Passive	12.99 ± 1.12
**Exposure To Tobacco Smoke**	
Exposure	12.91 ± 1.16
No Exposure	13.07 ± 1.25
**Mother’s Occupation**	
Housewife	13.05 ± 1.25
Employed	13.07 ± 1.21

^a^ A significant differences was observed between underweight group and the other three groups.

^b^ There were no data concerning girls who reached menarche between phase I and II; therefore, they were omitted.

**Table 3.** Multiple Regression Analysis of the Correlation Between Menarcheal Age and Various Variables^a^

Variables	Changes of Mean	95% CI	P. Value
**Maternal Menarcheal age**	0.66	0.58 to 0.73	< 0.001
**BMI**	-0.04	-0.06 to 0.01	0.002
**Maternal Educational Level**	0.25	0.03 to 0.47	0.027

^a^ Abbreviation: BMI, body mass index; CI, confidence interval.

## 5. Discussion

The present longitudinal study demonstrated that the mean menarcheal age in girl participants of a TLGS cohort was 13.06 ± 1.24 years. Factors contributing to the onset of menstruation were BMI, maternal education, and maternal age at menarche whereas there was no significant association between the daughters menarcheal age and mother’s occupation, exposure to tobacco smoke, physical activity, and calorie intake during premenarcheal years. In a survey of 10-16 year old girls (n = 370), living in 17th district of Tehran in 2009, mean menarcheal age was 12.6 ± 1.116 years. In another study of 580 female students in elementary, middle, and high schools also living in17th district of Tehran in 2001-2002, 88.1% of girls had menarche between 11 to 13 years of age, with a mean age of 12.1 ± 1.2 years (13). In another study, the median age at menarche for 6 to 17 year old girls in Tehran in 2001-2004 was 12.68 years (14). The mean of menarcheal age in 11 to 18 year old girls in Shahrekord city was 12.7 ± 1.15 years (15); in addition, in 24 provinces of Iran, the mean menarcheal age in girls born between 1983-1990 was 13.18 years (16). The difference between the mean onset of menstruation in the present study and other studies could be partly explained by the age range of our study participants (12-18 years) as girls who had their first period at a younger age were not included in this study. Thus, it appears that removal of this group of girls resulted in elimination of bias in the results.

The significant association observed at the onset of puberty in ethnic/regional groups and family members, indicates that the timing of puberty is regulated genetically as 50% to 80% of changes during this period are influenced by genetic factors (22). There was a positive correlation between maternal and daughters’ menarcheal age in our study, which was in accordance with the findings of Tehrani et al. (17) and Ainy et al. (18). Pouta also observed a significant positive correlation between mothers’ and daughters’ age at menarche (23).

In the present study, there was a significant inverse correlation between the age at menarche and BMI; however, when we compared the mean of menarcheal age according to the BMI categories, we found that menarcheal age of girls in the overweight group was lower than that underweight ones while those in the obese group had their menarche at later ages in comparison to the overweight group. This paradox can be explained by the fact that the fat stored in underweight girls in less than the critical amount of body fat (17%) needed for the onset of menstruation; therefore, their menarche occurs at later ages (24). On the other hand, obesity is an increase in fat storage, affecting the circulating levels of estrogen, androgen, and leptin; therefore, responsiveness of the hypothalamus-pituitary axis is impaired and menarche is delayed (24). A paradox also reported by Farahmand et al. (20), Bini et al. (25), and Onyiriuka et al(26); however, in a study conducted by Demerath et al., no association between age at menarche and BMI was observed (27). These contradictory findings could be attributed to differences in the geographic location, socioeconomic status, and the lifestyle (28, 29).

In accordance with the findings of the study by Padez et al. (29), we found a significant direct association between maternal educational level and the age at menarche; however, an inverse association was reported by Mollaei et al. in Gorgan (11) and Oduntan et al. in Nigeria (30). In the present study, there was no association between mother’s occupation and age at menarche, which was in agreement with the findings of Padez (29) and Danesh-Shahraki (15). It seems that maternal employment does not have any direct effect on menarcheal age and might exert its effect indirectly through influencing family lifestyle.

Prenatal exposure to nicotine affects fertility in offspring by altering production of sex hormones and gonadotropins (FSH and LH), all of which are key chemicals triggering the menarche (31). There are controversial opinions concerning the effect of exposure to tobacco smoke on menarcheal age; some studies demonstrate a significant association between maternal smoking during pregnancy and early menarcheal age of their daughters (30, 32). Another study, however, showed that maternal smoking during pregnancy and/or childhood exposure to environmental tobacco smoke would postpone menarcheal age of daughters (8). Similar to Shrestha et al. (31) and Fukuda et al. (33), we found no association between childhood exposure to the environmental tobacco smoke and menarcheal age.

It is reported that in addition to calories consumption, its expenditure is also important in onset of puberty; girls who have to do more physical work or have to go a long way to school have a greater expenditure of calories, which might delay the onset of puberty (34). After considering both physical activity and calorie intake, we found no association between the menarcheal age and these two variables, which is in accordance to the findings of the Bagga study (27). Merzenich et al. (10), however, observed a significant of physical activity and calorie intake with menarcheal age. Frisch et al. (35) and Vandenbroucke et al. (36) found that intense exercise could delay the onset of menarche; on the other hand, Mesaki et al. (37) showed that the mean physical activity was not associated with age at menarche. It seems that moderate physical activity and calorie intake do not directly influence the menarcheal age; instead, they act through changing BMI and stored body fat and consequently, the secondary effect on the hypothalamus-pituitary axis.

The main strength of the present study was its methodology; the prospective nature of this study allowed us to investigate the premenarcheal effects of environmental, socioeconomic, and individual factors on menarche timing. Our study had some limitations as well; we excluded females with menarche between phases I and II of the study due to the lack of measurements of MET and energy expenditure in phase I. Furthermore, considering measurements of MET and energy expenditure at phases II and III as a proxy for MET and energy expenditure at baseline, the lack of access to the hormonal profiles and the advanced methods for assessing obesity or calorie expenditure can also considered as a limitation to the present study. In addition, as we considered a wide range of ages at the baseline; age might have had an influence on BMI while the opposite is not true. Moreover, the prospective design of our study would be preferred to a cross-sectional design with no discrimination between exposure and outcome.

In conclusion we found that menarcheal age was influenced by premenarcheal BMI, maternal age at menarche, and maternal education, factors among which premenarcheal BMI is modifiable and maintaining an appropriate BMI could hence prevent early or late menarche.
